# Critical window of gestational greenspace exposure for the risk of low birth weight

**DOI:** 10.1088/1748-9326/adf86b

**Published:** 2025-08-15

**Authors:** Seulkee Heo, Kelvin C Fong, Ji-Young Son, Michelle L Bell

**Affiliations:** 1 School of the Environment, Yale University, New Haven, CT 06511, United States of America; 2 Milken Institute School of Public Health, The George Washington University, Washington, DC 20052, United States of America

**Keywords:** greenspace, vegetation, low birth weight, pregnancy trimesters, infant

## Abstract

Many studies link average residential greenspace exposure during pregnancy to birthweight changes, but evidence on critical timing for low birthweight is limited. Furthermore, coarse aggregations of exposure levels throughout pregnancy may obscure complex exposure-response relationships. This case-control study using the birth data (*n* = 788,275) in three US states examined the associations between the ZIP code-level weekly enhanced vegetation index (EVI) levels during gestational weeks 0–39 and term low birthweight (TLBW). The logistic regression with distributed lag non-linear functions, adjusted for maternal characteristics and season, estimated odds ratios (OR) of TLBW per interquartile range increase (0.200) in weekly EVI. Week-specific ORs showed an inverted U-shape. Significant ORs were observed in weeks 0–7 and 30–39, ranging from 0.989 (95% CI: 0.978–0.999) to 0.996 (95% CI: 0.992–1.000). Results highlight the importance of higher greenspace exposure in early and late pregnancy for reducing TLBW risk, informing policy and future research.

## 1. Introduction

Vegetation indices are commonly used to assess exposure to greenspace (land areas with vegetation) or greenness (amount of vegetation) (Mizen *et al* 2024). Vegetation indices, such as the normalized difference vegetation index (NDVI), are derived from satellite imagery based on light reflection and absorption by vegetation. Studies have examined greenspace’s impact on birth outcomes, including birth weight, term low birth weight (TLBW), and preterm birth (Hu *et al* 2021). Proposed biological mechanisms include mitigation of environmental hazards (e.g. heat island effect), psychological restoration, promotion of physical activity, and social cohesion (Hu *et al* 2021). However, epidemiologic findings remain inconsistent. While many NDVI studies report reduced risks of low birthweight with higher greenspace exposure (Dzhambov *et al* 2019, Nieuwenhuijsen *et al* 2019, Seabrook *et al* 2019), some found null associations (Abelt and McLafferty 2017, Yin 2019). Additionally, the enhanced vegetation index (EVI) improves upon NDVI by correcting distortions from aerosols, ground cover, and chlorophyll saturation. However, few studies have assessed EVI’s impact on birth outcomes (Ye *et al* 2023).

Studies of pregnancy outcomes commonly consider exposure windows for environmental exposures of whole pregnancy and trimesters. The critical exposure window for associations between greenspace and birth outcomes varied across studies; some suggested the highest increase in *z*-score birthweight per unit increase in NDVI during the whole pregnancy (Yang *et al* 2024), while others suggested higher associations between birthweight and NDVI exposures during the second trimester (Ye *et al* 2023). However, trimester averages may not fully capture associations when biological changes do not align with strict 3 month intervals (Wilson *et al* 2017). Trimester-specific analysis may also limit examination of potential lags between exposure and outcomes and temporal correlation between exposure and covariates. Emerging research explores environmental exposures at finer temporal scales, such as weekly intervals, offering improved insight into fetal health impacts. This approach is increasingly used for gestational air pollution and heat exposure (Sheridan *et al* 2019, Nyadanu *et al* 2023). However, to our knowledge, no studies have examined week-specific greenspace effects on birth outcomes despite the need to identify critical exposure windows and enhance care strategies for pregnant women accordingly.

Understanding temporally resolved greenspace exposure is critical, as it varies by location and season. Utilizing EVI data could enhance knowledge of how greenspace influences fetal development. We investigated critical gestational weeks for greenspace exposure and TLBW using EVI and population-based birth data from three US states. Weekly analyses were compared to trimester-based estimates. The findings can guide prenatal care and environmental policies by aiding our understanding of greenspace’s impact on fetal development and its critical exposure periods.

## 2. Methods

### 2.1. Data

This case-control study used birth certificate data for singleton births in Connecticut (2016–2018), New Jersey (2016–2020), and Pennsylvania (2016–2020). The outcome, TLBW, was defined as <2500 g for full-term births (⩾37 gestational weeks). We included 788 275 singleton births of mothers residing in the study states and with available gestational age, birthweight, and covariates (figure S1).

We obtained daily EVI data from MODIS Terra (MOD09GA v6.1), which are raster data (500 m for pixel sizes) with values ranging from –1 to 1. Higher values indicate denser vegetation and negative values indicate water, clouds, or bare soil. As we only had ZIP code information for participants’ residences (i.e. no point-level addresses), we calculated area-weighted average EVI within each ZIP code by averaging pixel values that fell within or overlapped with the ZIP code boundaries. We assigned weekly (7 d average) EVI levels for each birth from the last menstrual period to delivery date based on gestational age and the maternal residential ZIP code at delivery. Trimester-specific EVI exposure was also estimated by averaging daily EVI values for the first (weeks 0–13), second (weeks 14–26), and third (weeks 27–birth) trimesters.

Air pollution and ambient temperature exposures during pregnancy were estimated using ZIP code-level PM_2.5_ and modeled temperature data (Hammer *et al* 2020, PRISM Climate Group 2022). The PM_2.5_ estimates were derived by integrating satellite-based aerosol optical depth retrievals, chemical transport model, and ground-based monitoring data using geographically weighted regression, with a spatiotemporal resolution of approximately 1 km and monthly averages. As PM_2.5_ estimates were not available at a weekly basis, we considered the average PM_2.5_ levels over the whole pregnancy for each participant in our risk estimation model. The health effects of greenspace may vary by urbanicity due to variation in the dominant types of greenspace (Xiao *et al* 2022). Urban/non-urban ZIP codes in our study were classified using 2020 Census Urban Areas data (Census Bureau 2021).

### 2.2. Statistical analysis

We used logistic regression with a distributed lag non-linear model (DLNM) (Nyadanu *et al* 2023). DLNM minimizes temporal collinearity by smoothing exposure effects at adjacent lags (Mork and Wilson 2022). A bidimensional cross-basis matrix captured weekly EVI exposure effects on TLBW from the week of the last menstrual period to the 39th gestation week (i.e. 0–39 gestational weeks), with truncation at 39 weeks and post-delivery exposures set to zero. Setting the exposures to zero after the event (i.e. delivery) ensures that the exposure at the time of the event (i.e. the week of delivery) is treated as the instantaneous effect. Therefore, the cumulative lagged effect reflects only the exposures that occurred prior to the event (i.e. delivery).

To model the exposure-response relationship in the DLNM, we first compared two approaches: the linear exposure-response relationship model and the non-linear exposure-response relationship model. This non-linear function was modeled with three degrees of freedom (df) for the exposure-response relationship and the lag structure using a natural cubic spline. The resulted bi-dimensional curve plots of the exposure-response and lag-specific relationship curves from the second model exhibited a similar pattern to those from the first model, indicating a nearly linear exposure-response relationship (figure S2). Therefore, the exposure-response relationship was modeled with a linear function. In the selected linear exposure-response relationship DLNM, we modeled the lag structure using a natural cubic spline with 3 df, selected based on Akaike Information Criteria comparisons (df = 3, 4, 5). Models adjusted maternal and infant covariates pre-selected via a directed acyclic graph (figure S3) and literature (Hu *et al* 2021): season of conception, maternal age, infant sex, maternal smoking, maternal race/ethnicity, parity, maternal education (high school or less, some college or college graduated, and post-graduate degree), gestational age, prenatal care, urbanicity (urban vs. rural), residential state, weekly ambient temperature, average PM_2.5_ during pregnancy, and community-level socioeconomic status. While the directed acyclic graph may not capture all possible pathways, it illustrates our key assumptions (e.g. gestational age and temperature as a confounder). We controlled for the average PM_2.5_ exposure over whole pregnancy as weekly PM_2.5_ data were not available. We adjusted for gestational age to account for variability in pregnancy duration within term births as gestational age is correlated with birth weight and may be influenced by greenspace exposure (Grazuleviciene *et al* 2015). We adjusted for temperature effects to disentangle its correlation with EVI and reduce lagged confounding. We used a cross-basis function with a cubic spline (df = 3) for weekly temperature effects and a non-linear lag structure (df = 3) across weeks 0–39. Exposure-response associations were expressed as odds ratios (OR) and 95% confidence intervals (CI) per interquartile range (IQR) increase in weekly EVI (0.200). To adjust for the community-level socioeconomic status, we used the area deprivation index (ADI), a composite measure of neighborhood socioeconomic disadvantage based on income, education, employment, and housing quality, where higher values indicate greater deprivation (Kind and Buckingham 2018). To align with our ZIP-code level exposures, we used tertile groups of within-state ADI, which ranks ZIP codes by socioeconomic disadvantage within each state.

We examined whether the use of exposure averages during trimesters in the model influences the identification of critical exposure windows. Trimester-specific associations were estimated using DLNM with stratified lag structures (gestational weeks 0–13, 14–26, 27–39). Additional stratified logistic models accounted for inter-trimester exposure correlations using adjusted EVI residuals as the exposure variable. Within each trimester model, EVI residuals, regressed on the EVI values from the other two trimesters, were included as confounders to prevent covariance among exposure variables across trimesters (Bell *et al* 2007).

In our sensitivity analysis, we used average temperature exposure during pregnancy instead of its cross-basis to examine model robustness. While the DLNMs offer a flexible framework to simultaneously model lagged and non-linear effects of multiple exposures, the inclusion of multiple cross-basis functions in a DLNM for highly correlated exposures may introduce bias in risk estimates (Liao 2017). Replacing weekly temperature with the average temperature over the whole pregnancy could remain the hypothesized biological mechanisms of temperature, reduce the computational complexity of the DLNM, and evaluate the robustness of our results to the inclusion of multiple cross-basis functions.

We conducted another sensitivity analysis for the weekly DLNM that further adjusted for temporal trends of the date of conception. In addition to season, this model used a natural cubic spline for date of conception capturing finer temporal trends as fetuses conceived in different months may differ in characteristics not fully explained by seasonal categories.

## 3. Results

Of the 788 275 included infants, 18 823 were TLBW (2.4%) (table 1). Average EVI exposure levels during pregnancy were 0.169 (standard deviation [SD] = 0.062) for TLBW and 0.180 (SD = 0.063) for non-TLBW (table 1(A)). EVI levels peaked in summer, while participants’ average exposure peaked near the 40th gestational week (figure S4). Pairwise Pearson correlation coefficients between EVI exposures were 0.18 between the 1st and 2nd trimesters, −0.45 between the 1st and 3rd trimesters, and 0.15 between the 2nd and 3rd trimesters. Descriptive statistics of average EVI exposure over the full pregnancy period, stratified by maternal education and area-level deprivation, indicated lower vegetation exposure among individuals with lower socioeconomic status (table S1).

**Table 1.** Descriptive statistics of infant births (*n* = 788 275).

	*N* (%)		
	TLBW (Yes)	TLBW (No)	All
All	18 823 (2.4)	769 452 (97.6)	788 275
Infant sex			
Male	7492 (1.9)	394 369 (98.1)	401 861
Female	11 331 (2.9)	375 083 (97.1)	386 414
Infant race/ethnicity			
Non-Hispanic white	7,091 (1.8)	391 944 (98.2)	399 035
Non-Hispanic black	4280 (4.0)	104 007 (96.0)	108 287
Hispanic	4532 (2.3)	193 379 (97.7)	197 911
Asian	1822 (3.7)	47 521 (96.3)	49 343
Other	1098 (3.3)	32 601 (96.7)	33 699
Parity			
Nulliparous	9312 (3.0)	296 707 (97.0)	306 019
Multiparous	9511 (2.0)	472 745 (98.0)	482 245
Mother’s smoking during pregnancy			
No	15 707 (2.2)	712 072 (97.8)	727 779
Yes	3116 (5.2)	57 380 (94.8)	60 496
Receiving prenatal care			
No	235 (4.6)	4,909 (95.4)	5,144
Yes	18 588 (2.4)	764 543 (97.6)	783 131
Maternal education level			
⩽High school education	8332 (3.0)	268 847 (97.0)	277 179
College (received or graduated)	7899 (2.1)	364 238 (97.9)	372 137
Post-graduate degree	2592 (1.9)	136 367 (98.1)	138 959
Season of conception			
Spring	4724 (2.5)	184 324 (97.5)	189 048
Summer	4573 (2.3)	192 560 (97.7)	197 133
Fall	4694 (2.3)	200 626 (97.7)	205 320
Winter	4832 (2.5)	191 942 (97.5)	196 774
Urbanicity			
Urban ZIP codes	9304 (2.7)	336 936 (97.3)	346 240
Non-urban ZIP codes	9519 (2.2)	432 516 (97.8)	442 035
Enhanced vegetation index (EVI)			
1st trimester (mean ± SD)	0.185 (0.127)	0.193 (0.130)	0.194 (0.130)
2nd trimester (mean ± SD)	0.190 (0.129)	0.193 (0.130)	0.193 (0.130)
3rd trimester (mean ± SD)	0.161 (0.138)	0.184 (0.137)	0.183 (0.137)
Whole pregnancy (mean ± SD)	0.178 (0.132)	0.190 (0.133)	0.190 (0.133)
Ambient temperature (°C)			
1st trimester (mean ± SD)	12.0 (9.4)	11.9 (9.4)	11.9 (9.4)
2nd trimester (mean ± SD)	12.1 (9.5)	11.7 (9.4)	11.7 (9.4)
3rd trimester (mean ± SD)	10.5 (9.7)	11.3 (9.6)	11.3 (9.6)
Whole pregnancy (mean ± SD)	11.5 (9.6)	11.6 (9.5)	11.6 (9.5)
PM_2.5_ (*μ*g m^−3^) during whole pregnancy (mean ± SD)	7.4 (0.9)	7.3 (0.8)	7.3 (0.8)

**Figure 1.**
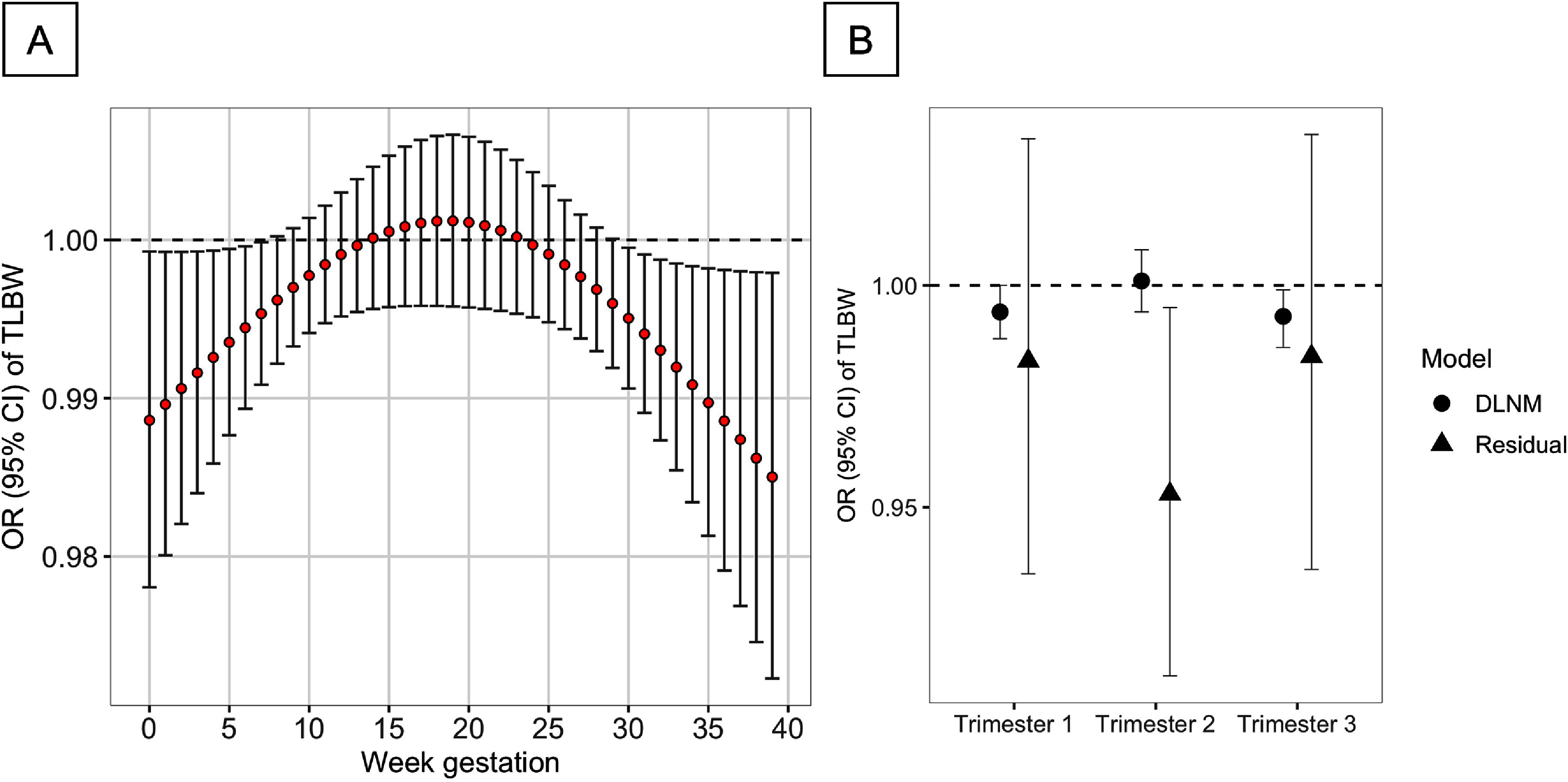
(A) Lag-specific odds ratios (ORs) of term low birthweight (TLBW) for an interquartile range increase in the enhanced vegetation index (EVI) exposure (0.200) and (B) trimester-specific ORs. The solid dots represent the OR and the lines indicate the corresponding 95% confidence intervals in Figure (A). The symbol ‘residual’ represents the results from the trimester-specific regression models adjusting the correlations of EVI values across trimesters.

The lag-specific ORs showed an inverted U-shaped curve with significant associations during 0–7th and 30–39th weeks of gestation (figure 1). The OR for LBW, for a 0.200 increase in EVI, was 0.989 (95% CI: 0.978–0.999) in gestational week 0 and 0.995 (95% CI: 0.991–1.000) in the 7th gestational week. The OR was 0.995 (95% CI: 0.991–1.000) and 0.985 (95% CI: 0.972–0.998) during the 30th and 39th weeks, respectively. The trimester-specific OR derived from the DLNM was 0.994 (95% CI: 0.988–1.000), 1.001 (95% CI: 0.994–1.008), and 0.993 (95% CI: 0.986–0.999), respectively (figure 1(B)). The trimester-specific ORs for the correlation-adjusted EVI averages were 0.983 (95% CI: 0.935–1.033) in the first trimester, 0.953 (95% CI: 0.912–0.995) in the second trimester, and 0.984 (95% CI: 0.936–1.034) in the third trimester (figure 1(B)). The difference in log OR across trimesters was not significant (appendix).

In the sensitivity analysis using average pregnancy temperature instead of weekly values, ORs for EVI for weeks 30–39 remained robust, while those for gestational weeks 0–7 slightly shifted away from the null, extending the critical window to weeks 0–12 (figure S5).

Other sensitivity analyses adjusting for date of conception and season showed robust results while weekly ORs shifted toward the null, with critical windows slightly shifting to weeks 0–6 and 30–39 (figure S6).

## 4. Discussion

No consensus exists on the most critical windows during pregnancy for greenspace exposure’s influence on low birthweight. We adopted DLNMs, effective in modeling lagged effects and correlations of environmental exposures on outcomes, and found greenspace exposure during gestational weeks 0–7 and 30–39 was associated with reduced TLBW risk at birth. Our DLNM with lag strata matching with trimesters showed similar results.

On the contrary, our regressions using trimester-specific exposure averages showed EVI’s impact during the second trimester. The DLNM with weekly exposures examines narrow susceptibility periods and temporal precision, while the model with trimester averages provides easier clinical interpretation. The differences in the critical exposure windows between the two models can be partially explained by the loss of information on exposure variability in the model using trimester-averaged values. Averaging greenness for a trimester can reduce week-to-week variation in the exposure and result in smoothed exposure levels with less variation. By aggregating these weekly values into trimester-specific averages, this temporal variation becomes compressed, potentially obscuring the variance in exposure levels and exposure timing. Furthermore, even if weekly EVI changes at a given ZIP code are small, differences in the gestational period across participants (e.g. gestation occurring primarily during the summer and another during the winter) can introduce meaningful inter-individual variation in the assigned weekly EVI exposure values. These trimester-specific averaged exposure values may improve the model’s ability to detect an association by reducing random fluctuations in weekly exposures. However, the temporal smoothing of exposures over a trimester can blur the timing of effects. Particularly, critical exposures occurring near the boundary of two trimesters may shift the apparent effect into an adjacent trimester and obscure more precise windows of vulnerability identified in weekly models. Placentation during early pregnancy and fetal growth from the second trimester can occur in narrow biological windows and therefore the week-level DLNM would be suitable to detect critical exposure windows. Our results highlight the need for careful evidence comparison as estimates for the association between greenspace and birthweight may differ depending on the temporal scale of exposure, whether based on trimester averages or weekly estimates.

Identifying critical exposure windows helps reveal how greenspace may influence fetal growth through stage-specific mechanisms. The greenspace effect in early pregnancy suggests a vital role in placental development and embryogenesis (Moore 2016). Effects observed in the second trimester would likely support fetal maturation and organ development, while those in late pregnancy would be important for rapid fetal weight gain and growth (Moore 2016).

To our knowledge, this study is the first to examine weekly lagged EVI exposure during pregnancy and TLBW. Using daily 500 m resolution EVI data improves spatiotemporal accuracy over 8- or 16 d composites, enabling precise weekly exposure assessment. We analyzed a large sample across three US states, utilizing the individual-level birth data. A limitation is that our data lacked some key covariates (maternal pre-pregnancy BMI, alcohol use, marital status, and health conditions such as gestational hypertension). However, adjusting for tobacco use may partially account for related maternal risk factors. Furthermore, the adjustment of individual-level and area-level socioeconomic status may partially adjust for these unmeasured covariates. Nonetheless, the lack of adjustment for key individual-level risk factors may influence the estimated effects of greenspace, and more rigorous adjustment should be considered in future research. Also, while conditioning on full-term births allowed us to focus on TLBW, it may introduce unmeasurable selection bias by excluding preterm births related to fetal growth restriction. Next, we assumed no address changes due to lack of data on residential mobility, which may introduce exposure measurement error. Information on residence duration was available for Connecticut but not for Pennsylvania and New Jersey, limiting our ability for more precise exposure assessments. However, prior research suggests that pregnant individuals typically move short distances, likely resulting in minimal impact on exposure estimates depending on spatial exposure variability (Bell *et al* 2018). Also, the temporal scale of our adjustment for PM_2.5_ during the whole pregnancy was coarse. Due to the temporal resolution of the obtained PM_2.5_ data (i.e. monthly), we could not consider weekly PM_2.5_ exposures, which would often exhibit substantial variance at a finer temporal scale (e.g. weekly). Given the complex correlations between atmospheric factors (e.g. air pollution and ambient temperature), the impact of differing temporal scales of PM_2.5_ as a confounder may be a valuable topic for future research. Other air pollutants likely impact birthweight, but due to data limitations, we could not include them in our models, requiring cautious interpretation. Future research is needed on the impacts by type of greenspace (e.g. park, crops).

In summary, we identified critical windows shorter than the 3 month trimester intervals examined in previous studies. Our findings emphasize the importance of greenspace exposure during the early and late stages of pregnancy. The results have implications for health policies for pregnancy through urban planning that ensures improved greenspace accessibility for populations and community-based family health programs offering education and activities. The findings may be informative in medical practices supporting advice about environment and consultations on the importance of greenspace exposure during pregnancy. Further research is needed to investigate how critical greenspace exposure windows may vary by region and confounder assessment strategies.

## Data Availability

The data cannot be made publicly available upon publication because they are owned by a third party and the terms of use prevent public distribution. The data that support the findings of this study are available upon reasonable request from the authors.
